# Metabolomics reveals metabolite changes of patients with pulmonary arterial hypertension in China

**DOI:** 10.1111/jcmm.14937

**Published:** 2020-01-16

**Authors:** Chenyang Chen, Fei Luo, Panyun Wu, Yiyuan Huang, Avash Das, Shenglan Chen, Jingyuan Chen, Xinqun Hu, Fei Li, Zhenfei Fang, Shenhua Zhou

**Affiliations:** ^1^ Department of Cardiovascular Medicine The Second Xiangya Hospital Central South University Changsha China; ^2^ Department of Cardiovascular Medicine The Third Xiangya Hospital Central South University Changsha China; ^3^ Department of Molecular Genetics University of Texas Southwestern Medical Center Dallas TX USA; ^4^ Kunming Institute of Botany Chinese Academy of Sciences Kunming China

**Keywords:** lipid metabolism, metabolomics, pulmonary arterial hypertension

## Abstract

The specific mechanism of pulmonary arterial hypertension (PAH) remains elusive. The present study aimed to explore the underlying mechanism of PAH through the identity of novel biomarkers for PAH using metabolomics approach. Serum samples from 40 patients with idiopathic PAH (IPAH), 20 patients with congenital heart disease‐associated PAH (CHD‐PAH) and 20 healthy controls were collected and analysed by ultra‐high‐performance liquid chromatography coupled with high‐resolution mass spectrometry (UPLC‐HRMS). Orthogonal partial least square‐discriminate analysis (OPLS‐DA) was applied to screen potential biomarkers. These results were validated in monocrotaline (MCT)‐induced PAH rat model. The OPLS‐DA model was successful in screening distinct metabolite signatures which distinguished IPAH and CHD‐PAH patients from healthy controls, respectively (26 and 15 metabolites). Unbiased analysis from OPLS‐DA identified 31 metabolites from PAH patients which were differentially regulated compared to the healthy controls. Our analysis showed dysregulation of the different metabolic pathways, including lipid metabolism, glucose metabolism, amino acid metabolism and phospholipid metabolism pathways in PAH patients compared to their healthy counterpart. Among these metabolites from dysregulated metabolic pathways, a panel of metabolites from lipid metabolism and fatty acid oxidation (lysophosphatidylcholine, phosphatidylcholine, perillic acid, palmitoleic acid, N‐acetylcholine‐d‐sphingomyelin, oleic acid, palmitic acid and 2‐Octenoylcarnitine metabolites) were found to have a close association with PAH. The results from the analysis of both real‐time quantitative PCR and Western blot showed that expression of LDHA, CD36, FASN, PDK1 GLUT1 and CPT‐1 in right heart/lung were significantly up‐regulated in MCT group than the control group.

## 1 INTRODUCTION

Pulmonary arterial hypertension (PAH) is a chronic disease condition involving in vascular remodelling disease of the lungs, which causes an increase in the pulmonary artery pressure and eventually leading to right heart failure and death. Despite the progress in knowledge has been obtained about potential therapeutic targets and introduction of newer drugs, the 5‐year survival rate still remains low.^1^ This can be attributed to the ill‐understood pathophysiology of PAH, necessitating further exploration of the mechanism of disease development for the development of novel therapeutic strategies.

In recent years, the major efforts have been focused on the differential regulation in the metabolic pathways which may be contributory to PAH pathogenesis. Emerging evidence points towards a metabolic theory of PAH, suggesting that PAH results from the suppression of the mitochondria‐based respiration and glucose oxidation (named Warburg effect in cancer metabolism).^2^, ^3^ It causes cells to rapidly proliferate without undergoing apoptosis of cells and accelerates the vascular remodelling in PAH. It is widely regarded that various metabolic changes during the Warburg effect are also essential for the occurrence and maintenance of PAH.^3^, ^4^ In addition, metabolic changes involving fatty acid oxidation and amino acid breakdown are also thought to be involved in the formation of PAH.^5^ A deeper understanding of these pathways holds the potential to provide targets for the diagnosis and treatment of PAH. However, due to the complex nature of these metabolic pathways in PAH, a single metabolic pathway affecting the disease pathogenesis and progression is difficult to illustrate. However, large scale metabolomics provides a lucrative alternative to understand the differential regulation of these metabolic pathways involved in PAH. In a seminal study by Zhao et al,^6^ the metabolomic heterogeneity of PAH patients was demonstrated in a discovery cohort of a small group of patients (n = 8). Unbiased metabolomic profiles hinted towards possible disruption of the glycolytic pathway with concurrent increase in TCA cycle substrates and metabolites. Using a combination of high‐throughput liquid‐and‐gas‐chromatography‐based mass spectrometry, the changes in fatty acid oxidation were shown from the resultant metabolites and subsequent changes in arginine pathways were also demonstrated with increase in nitric oxide (NO) production and decreased arginine levels in the lung. Further validation of predicted targets using a panel of 105 circulating plasma metabolites in PAH patients confirmed the association of right ventricular‐pulmonary vascular dysfunction with circulating indoleamine 2,3‐dioxygenase (IDO)‐dependent tryptophan metabolites (TMs), tricarboxylic acid intermediates and purine metabolites.^7^ Reports from recent study are also congruent with these findings where they demonstrate a strong strength of association between patient survival and metabolic profiles in PAH.^8^

However, the metabolic profile of individuals is dependent on race, sex and dietary habits. Till now, no studies focusing on the metabolomic changes in a primarily Chinese PAH patient cohort have been documented. The current study aimed to fill this gap in knowledge in our study. Employing ultra‐high‐performance liquid chromatography coupled with high‐resolution mass spectrometry (UPLC‐HRMS) in a non‐targeted metabolomics analysis, we aim to explore the metabolic profiles in plasma from PAH patients (idiopathic or congenital heart disease (CHD) associated PAH) and their healthy counterparts. We also investigated the differentially expressed targets in PAH rat model to delineate the changes in the key enzymes involved in glucose and lipid metabolism. This is intended to identify the signature of metabolites for PAH, which can be used for biomarker discovery and potential therapeutic targets for the disease germane.

## 2 MATERIALS AND METHODS

### 2.1 Sample collection

Samples were obtained from 40 patients with idiopathic PAH (IPAH) and 20 patients with CHD associated PAH (CHD‐PAH) at the Second Xiangya Hospital of Central South University, Changsha, China, between 2012 and 2015. Control plasma samples were obtained from 20 healthy volunteers. Pulmonary arterial hypertension was diagnosed by right‐side heart catheterization. The diagnosis of PAH was based on standard criteria from the 2015 ESC/ERS Guidelines for the Diagnosis and Treatment of Pulmonary Hypertension.^9^ All patients and healthy volunteers provided informed consent, and the study was approved by research ethics committees of the Second Xiangya Hospital of Central South University and was performed in accordance with the Declaration of Helsinki. Exclusion criteria consisted of the following: serious or uncontrolled chronic diseases such as severe cerebrovascular disease, malignant tumour, chronic severe lung disease (such as COPD) and chronic progressive nephropathy.

After fasting for 8 hours before right‐side heart catheterization, venous blood samples were drawn from the femoral venous and collected in EDTA anticoagulant tubes, centrifuged (1000 *g*, 10 minutes) and stored at −80°C until required.

### 2.2 Right‐side heart catheterization

Right‐side heart catheterization was performed by internal jugular vein. Haemodynamic measurements were investigated by a catheter as described previously.^10^ Ventricular systolic pressure (RVSP), right ventricular diastolic pressure (RVDP), right atrial pressure (RAP), mean pulmonary vein pressure (mPVP) and pulmonary capillary wedge pressure (PCWP) were documented. Pulmonary vessel resistance (PVR) = mPVP/pulmonary flow (L/min) was calculated.

### 2.3 Sample preparation and UPLC‐HRMS

The method of sample preparation for the metabolomic analysis was in accordance with the sample preparation method by UPLC‐HRMS.^11^ Briefly, serum samples were prepared by 10 µL serum mixed with 190 µL 67% aqueous acetonitrile. The samples were vortexed for 5 minutes and centrifuged at 18 400 *g* for 20 minutes at 4°C to remove particulates and precipitate protein. The supernatant was transferred to an autosampler vial for analysis.^12^ A 5 µL aliquot of supernatant samples was injected into the system of ultra‐performance liquid chromatography coupled quadrupole time‐of‐flight mass spectroscopy (UPLC‐ESI‐QTOFMS). The liquid chromatography system was ACQUITY UPLC® equipment (Waters) consisting of a reverse‐phase 2.1 × 50 mm ACQUITY UPLC® BEH C18 1.7 µm column (Waters Corp.) with a gradient mobile phase comprising 0.1% formic acid solution (A) and acetonitrile containing 0.1% formic acid solution (B). The gradient was maintained at 100% A for 0.5 minute, increased to 100% B over the next 7.5 minutes and returned to 100% A in last 2 minutes. Data were collected in positive mode and negative mode on a Waters Q‐TOF, which was operated in full‐scan mode at m/z 100‐1000. Nitrogen was used as both cone gas (50 L/h) and desolvation gas (600 L/h). Source temperature and desolvation temperature were set at 120°C and 350°C, respectively. The capillary voltage and cone voltage were 3000 and 20 V, respectively. Chlorpropamide (5 µmol/L) was added in the sample as the internal standard. A volume of 10 µL sample from each plasma ample was prepared as a quality control (QC) sample to validate the stability of sequence analysis. The QC sample was extracted and analysed in the same way as describe above. In order to evaluate the repeatability, QC sample and blank (pure acetonitrile) sample were injected after every 10 samples during the analytic run.

### 2.4 Data preprocessing and multivariate statistical analysis

Firstly, Profnder and Mass Profler Professional software were used to extract and analyse the original data. Then, the analysed data were imputed to SIMCA‐P+(13.0) software for multivariate pattern recognition analysis. We used a principal component analysis (PCA) to highlight potential outliers. Meanwhile, the quality control (QC) samples were analysed by PCA to detect the polymerization and the stability of the method. Orthogonal partial least square‐discriminate analysis (OPLS‐DA) was applied to the same data sheet to figure out the differences in metabolite in the groups. OPLS‐DA was also applied to figure out values of variable importance in projection (VIP) of each metabolite. Benjamini–Hochberg false discovery rate (FDR) is calculated based on the “BH” method.^13^ Student's *t* test used to compare the levels of metabolite in two independent group, where *P* values of <.05, VIP > 1.0 and FDR < 0.1 were considered to be significantly altered.

After preliminary screening of different molecules, the metabolites were identified by the following methods: (a) determine [M+H]^+^ and [M‐H]^−^ ions of the metabolites, and calculate the possible chemical composition according to their precise molecular weight; (b) looking for the possible structure for metabolites through high‐resolution MS and MS/MS spectrum analysis, comparing with online database (including MassProfnder source database, HMDB, HMDB serum, KEGG, MassBank, PubChem, etc); (c) screening with biological information; (d) comparing the retention time and mass spectrogram of the selected metabolites with the standard product.

### 2.5 PAH animal models

The protocol was approved by the Animal Research Committee, Central South University, Hunan, China, and carried out in accordance with the Guidelines for Animal Experimentation of Central South University and the Guide for the Care and Use of Laboratory Animals published by the US National Institutes of Health (NIH Publication No. 85‐23, revised 2011).

Male SD rats (180 g) were obtained from the Hunan SJA Laboratory Animal Co. Rats randomly received an intraperitoneal injection of normal saline (control, n = 12) or monocrotaline (MCT) (Sigma, 60 mg kg^−1^·rat, n = 24) to induce PAH. The rats in control group were examined at the third week (day 21), and rats in MCT group were randomly examined at the second (day 14, n = 12) and third week (day 21, n = 12).

### 2.6 Measurement of RVSP and RVH

Haemodynamic measurement was performed as previously described.^14^ Rats were anaesthetized by intraperitoneal injection of pentobarbital sodium. A venous catheter was inserted in the right jugular vein and introduced in the right atrium (RA) of rats to observe the RVSP. After haemodynamic measurement, the lungs and hearts were harvested. The RV and left ventricular (LV) plus interventricular septum (S) were separated and then weighed, respectively. Right ventricular hypertrophy (RVH) was using the ratio of RV weight to LV plus S weight [RV/(LV + S)].

### 2.7 Total RNA preparation and real‐time quantitative PCR analysis

Quantitative real‐time PCR was performed as previously described.^15^ Total RNA was extracted from lungs or heart tissue using TRIzol (Invitrogen) according to the manufacturer's instructions. The RNA was reverse‐transcribed to cDNA using a RevertAid First Strand cDNA Synthesis Kit (Thermo Scientific) and was performed with gene‐specific primers and SYBR Green Master Mix (Applied Biosystems). The data were normalized to GAPDH. The transcripts were assessed by real‐time PCR on a 7300 qPCR system (all from Applied Biosystems). Relative gene expression was quantified using the comparative threshold cycle value (∆CT) method with the above primers. The relative gene expression was calculated as fold change = 2^−∆(∆Ct)^.

### 2.8 Western blotting analysis

Western blotting analysis was performed as previously described.^16^ Briefly, the proteins from harvested lung were extracted with RIPA lysis buffer (Beyotime) and separated on sodium dodecyl sulphate‐polyacrylamide gel electrophoresis (SDS‐PAGE) before transferring onto polyvinylidene difluoride (PVDF) membranes. Subsequently, the membranes were blocked with 5% milk PBST solution and then incubated with the following respective antibodies: rabbit monoclonal anti‐CD36, rabbit monoclonal anti‐GLUT1, rabbit monoclonal anti‐PKM2 (Abcam), mouse monoclonal anti‐HIF‐1α, rabbit polyclonal anti‐FASN, rabbit polyclonal anti‐PPAR‐α, rabbit polyclonal anti‐PGC1 α (Novus), mouse monoclonal anti‐LDHA, mouse monoclonal anti‐PDK1, mouse monoclonal anti‐PDK4 (Proteintech), mouse monoclonal anti‐CPT‐1β (LSBio) or β‐actin control (Bios), at 4°C for overnight. Blots were washed, incubated with the secondary antibody (Proteintech) and visualized by chemiluminescence.

### 2.9 Statistical analysis

Comparisons between multiple groups were conducted using one‐way analysis of variance (ANOVA) followed by least significant difference post hoc tests. Comparisons between two groups were conducted using Student's *t* test. The difference with *P* < .05 (two sides) was statistically significant. Potential biochemical markers were further evaluated by receiver operating characteristic (ROC) analysis. The area under the ROC curve (AUC) is to assess the sensitivity and specificity of the biomarkers. AUC values >0.9 indicate high reliability of the model, 0.7‐0.9 indicate moderate reliability, 0.5‐0.7 indicate poor reliability and AUC ≤ 0.5 suggests that the model prediction is not better than chance.

## 3 RESULTS

### 3.1 Basic information of participants

The average age of IPAH patients and CHD‐PAH patients was 37.72 ± 10.50 and 31.65 ± 8.29 years with a female predominance (Table 1). The CHD‐PAH patients were consisted of ventricular septal defect (55%), atrial septal defect (25%) and patent ductus arteriosus (20%).

**Table 1:** Basic information of participants

	IPAH (n = 40)	CHD‐PAH (n = 20)	Healthy control (n = 20)
Age (y)	37.72 ± 10.50	31.65 ± 8.29	34.15 ± 3.56
Female (n, %)	29 (72.5%)	13 (65%)	11 (55%)
BMI (kg/m^2^)	22.03 ± 4.45	18.10 ± 3.61	21.70 ± 2.09
Chronic heart failure (NYHA I or II)	24 (60%)	14 (70%)	/
Chronic heart failure (NYHA III or IV)	16 (40%)	6 (30%)	/
Acute pulmonary vasodilator responder	4 (10%)	2 (10%)	/

### 3.2 Clinical characteristics

The levels of NT‐proBNP, uric acid and total bilirubin in IPAH patients were significantly higher than normal and only the levels of total bilirubin in CHD‐PAH patients were significantly higher than normal value (Table 2). The results of cardiac colour ultrasound showed the diameter of right ventricle (RV) and right atrium in IPAH patients was larger than normal value. The diameter of RV in CHD‐PAH patients was larger than normal value (Table 2). The results of right‐side heart catheterization showed RVSP, RVDP, mPVP and pulmonary vessel resistance were significantly higher than normal value both in IPAH and CHD‐PAH patients (Table 2).

**Table 2:** Clinical characteristics of participants

Biochemistry (fasting)	IPAH (n = 40)	CHD‐PAH (n = 20)
Creatinine μmol/L	71.74 ± 21.3	21.04 ± 29.73
Uric acid μmol/L	436.59 ± 108.43	335.62 ± 65.51
Blood urea nitrogen mmol/L	5.49 ± 2.02	2.30 ± 1.94
NT‐proBNP pg/mL	2272.42 ± 2352.6	158.85 ± 229.9
ALT u/L	37.48 ± 56.6	35.61 ± 15.21
AST u/L	37.73 ± 38.42	17.3 ± 7.91
Total bilirubin μmol/L	19.7 ± 9.7	27.51 ± 27.74
Cardiac colour ultrasound		
RA (mm)	49.19 ± 11.22 (25‐85)	39.07 ± 8.87 (30‐60)
RV (mm)	45.52 ± 10.66 (20‐60)	39.27 ± 5.24 (30‐50)
LA (mm)	33.55 ± 7.21 (18‐46)	44.53 ± 14.6 (24‐84)
LV (mm)	27.67 ± 4.55 (17‐38)	31.67 ± 8.8 (20‐50)
PA (mm)	32.0 ± 12.86 (24‐90)	34.33 ± 7.91 (21‐50)
Tricuspid regurgitation velocity (m/s)	5.8 ± 4.0 (0‐5.8)	4.27 ± 0.95 (2‐4.9)
Pulmonary valve reflux velocity (m/s)	2.07 ± 1.08 (0.48‐3.8)	3.45 ± 0.96 (2‐4.4)
EF (%)	63.18 ± 7.83 (35‐74.5)	58.73 ± 16.4 (22‐84)
Haemodynamics characteristics		
RVSP (mm Hg)	93.27 ± 21.67 (52‐132)	108.38 ± 19.38 (69‐144)
RVDP (mm Hg)	43 ± 10.23 (−4 to 43)	53 ± 10.89 (29‐68)
mPVP (mm Hg)	60.38 ± 14.65 (38‐92)	47 ± 8.79 (27‐59)
RAP (mm Hg)	13.27 ± 6.69 (0‐24)	11.88 ± 3.63 (6‐19)
Qp/Qs	/	1.28 ± 0.48 (0.7‐2.1)
Pulmonary vascular resistance, Woods units	19.40 ± 10.4 (7.04‐53.57)	14.95 ± 4.86 (8.32‐22.8)

### 3.3 Metabolomic profiles

The results of PCA showed there were no remarkable outliers, and the stability of the method was high (data not shown). Mass Profnder and Mass Profler Professional software were used to extract the raw data and conducted peak extraction, the peak cluster as well as retention time correction. Ultimately, we got the data about mass‐to‐charge ratio (m/z), the retention time (RT) and the data matrix of peak area. In positive and negative ion mode, 1175 and 907 variables were obtained, respectively.

**Figure 1:**
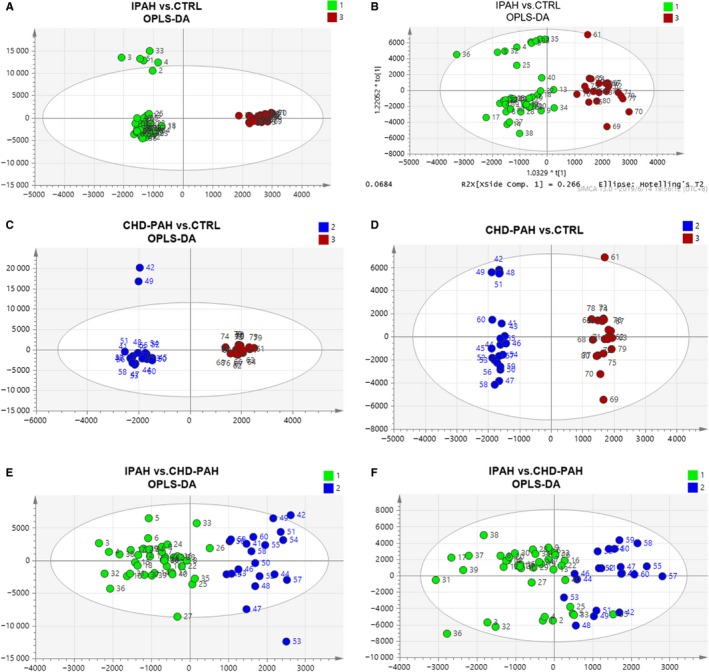
OPLS‐DA score plots based on metabolites/peak areas. Scores plot generated from OPLS‐DA model shows IPAH (green) as compared with the control (red) in positive ion model (A) and negative ion model (B), CHD‐PAH (blue) as compared with the control (red) in positive ion model (C) and negative ion model (D), IPAH (green) as compared with the CHD‐PAH (blue) in positive ion model (E) and negative ion model (F)

OPLS‐DA was used to analyse the different metabolites in positive and negative ion mode. The results showed a clear separation in model 1 (IPAH vs Control) (Figure 1A,B), model 2 (CHD‐PAH vs Control) (Figure 1C,D) and model 3 (IPAH vs CHD‐PAH) (Figure 1E,F). The evaluation parameters of these models were obtained by cross validation of the OPLS‐DA model ten times, confirming the model's robust risk‐prediction ability (Table S1). And S‐plot model was applied to figure out the differential variable. When VIP value of the metabolite was greater than 1.0, it is considered as a significant contribution to the model. Student's *t* test was used to analyse the difference between two groups. Results showed that 144 (in positive ion model) and 67 (in negative ion model) differential variable distinguished IPAH patients from healthy controls. Fifty‐six (in positive ion model) and 50 (in negative ion model) differential variable distinguished CHD‐PAH patients from healthy controls. Twenty‐three (in positive ion model) and 26 (in negative ion model) differential variable distinguished IPAH patients from CHD‐PAH patients.

**Figure 2:**
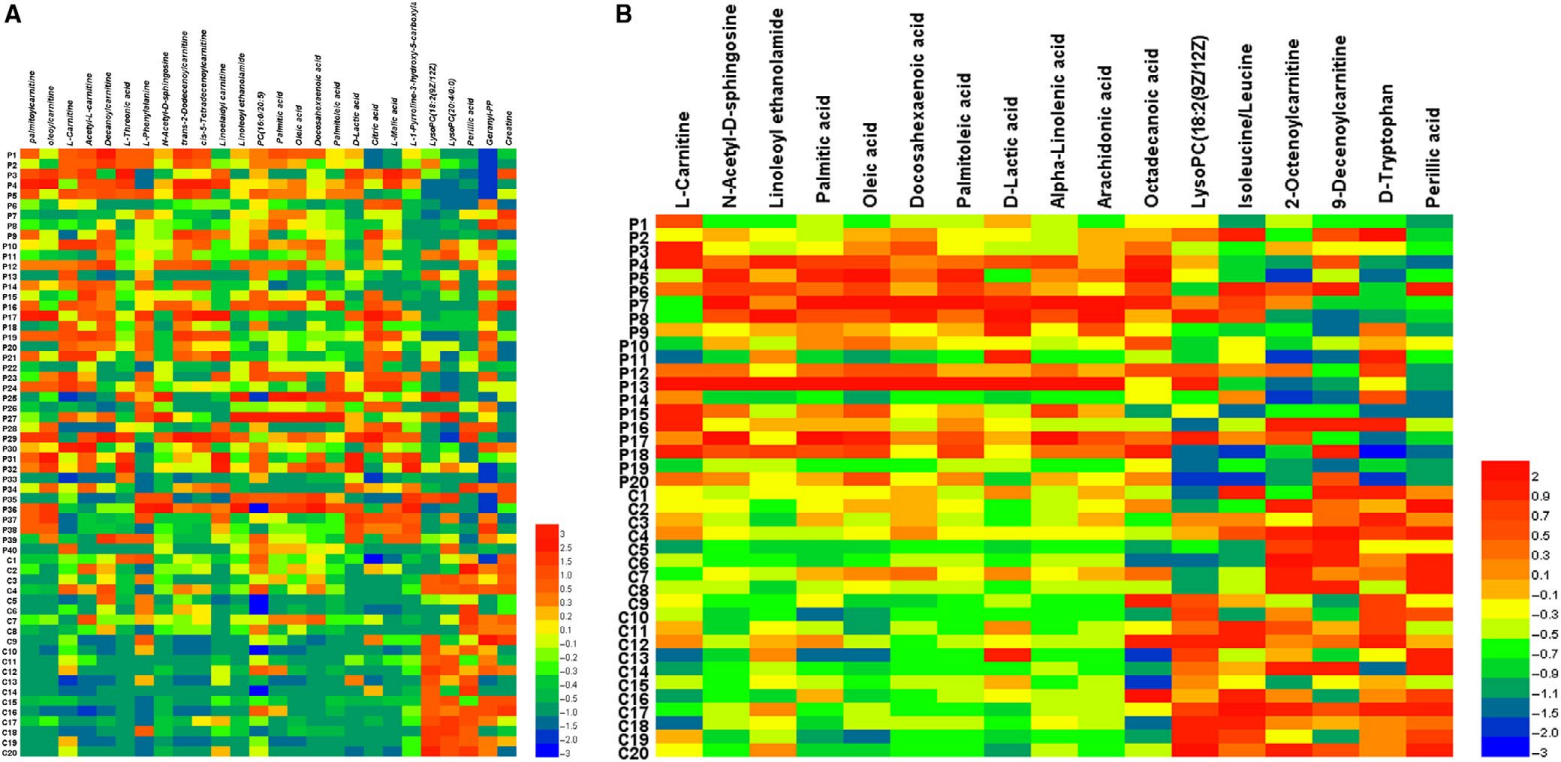
Heat map of differential metabolites. Heat map of 33 differential metabolites relative to IPAH sample (n = 40) data over Healthy control (CTRL, n = 20) (A), and CHD‐PAH sample (n = 20) data over Healthy control (CTRL, n = 20) (B). Shades of light red/blue represent the increase and decrease in a metabolite, respectively, other colours relative to the median metabolite levels

We finally found 26 and 15 metabolites distinguished IPAH or CHD‐PAH from healthy controls, respectively, including 18 positive ions and 13 negative ions (Table S2). Among these metabolites, 31 metabolites from PAH patients were significantly up‐regulated (n = 22) or down‐regulated (n = 9) compared with respective metabolites from healthy controls (Table S2, Figure 2A,B).

### 3.4 Enrichment and clustering of metabolites of interest

**Figure 3:**
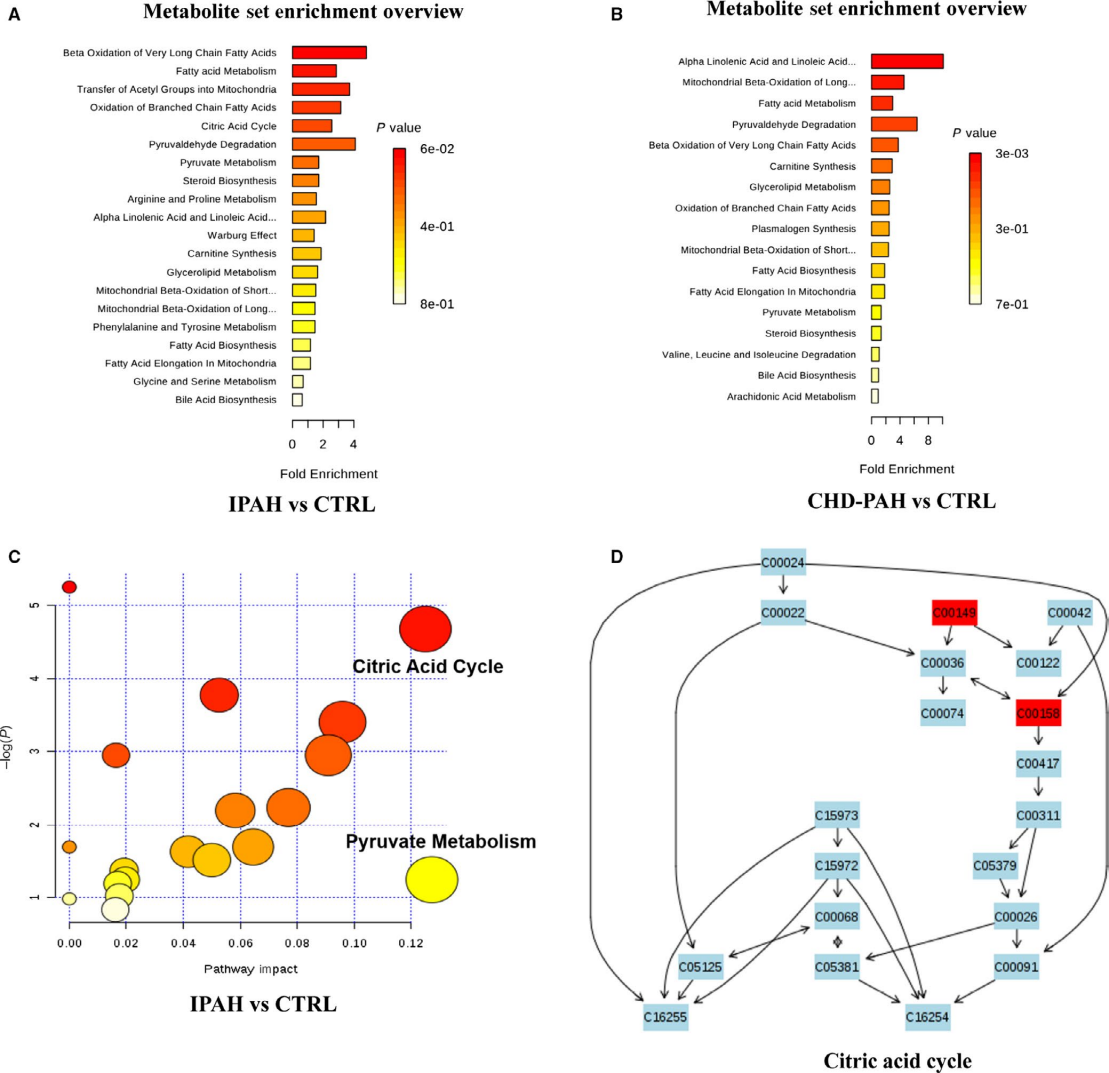
Enrichment overview and functional analysis of metabolic pathways. The metabolic pathway enrichment and clustering analysis showed the pathways enriched with metabolites of interest which distinguished the IPAH (A) or CHD‐PAH (B) from healthy controls (CTRL). IPAH group metabolic function bubble chart (C) and citric acid cycle metabolic pathway (malic acid and citric acid marked with red are significantly changed in peripheral blood plasma of IPAH patients) (D)

The metabolic pathway enrichment and clustering analysis showed 20 pathways were enriched with metabolites of interest which distinguished the IPAH and healthy controls (Figure 3A). Among these pathways, beta‐oxidation of very long‐chain fatty acids, fatty acid metabolism, transfer of acetyl groups into mitochondria, oxidation of branched‐chain fatty acids, citric acid cycle, pyruvaldehyde degradation and pyruvate metabolism showed the highest enrichment level (Figure 3A).

Seventeen pathways were enriched with metabolites of interest which distinguished the CHD‐PAH and healthy controls (Figure 3B). Among these pathways, alpha‐linolenic acid and linoleic acid metabolism, mitochondrial beta‐oxidation of long‐chain fatty acids, pyruvate decomposition, oxidation of branched‐chain fatty acids, and carnitine synthesis showed the highest enrichment level (Figure 3B).

Functional analysis of metabolic pathway showed citric acid cycle (impact 0.125) and phenylalanine metabolic pathway (impact 0.127) were remarkable disturbed in IPAH patients (Figure 3C). Malic acid and citric acid in citric acid cycle are significantly regulated in IPAH patients (Figure 3D). Alpha‐linolenic acid metabolism (impact 0.203) and arachidonic acid metabolism (impact 0.203) were remarkable disturbed in CHD‐PAH patients (Data not shown).

### 3.5 ROC curve analysis of metabolites

**Figure 4:**
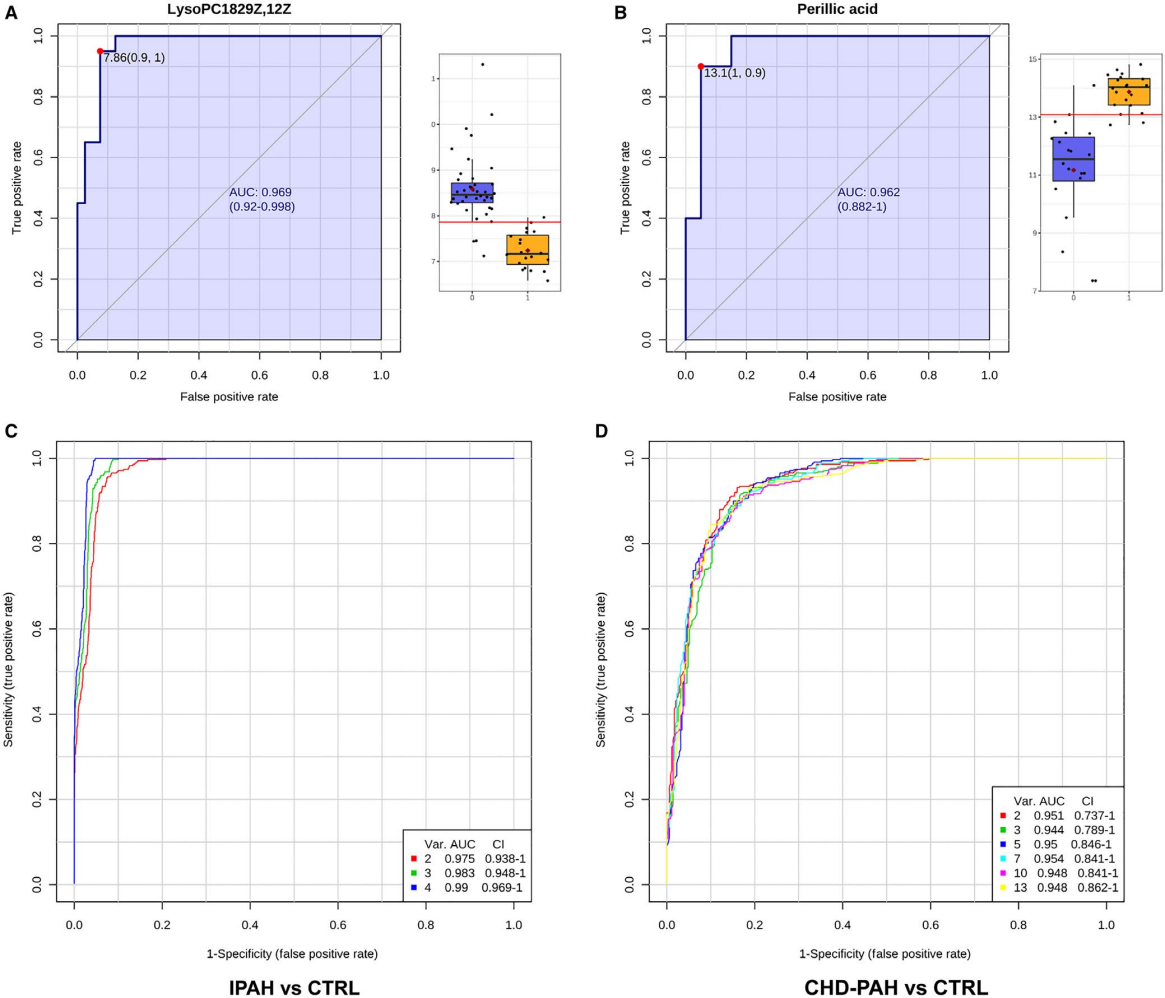
ROC curve. ROC curve of LysoPC (18:2(9Z,12Z) (A), and perillic acid (B). The joint diagnostic efficacy of selected metabolites was analysed by random forest method, the AUC for IPAH (C) or CHD‐PAH (D)

In order to assess the diagnostic capacity of the metabolites, the receiver operating characteristic (ROC) curve analysis was applied to the data sheet. The area under the ROC curve for LysoPC, PC, decanoylcarnitine and l‐carnitine was 97%, 83%, 79% and 74.4% in IPAH patients, respectively, which indicated LysoPC (18:2(9Z,12Z)) had a good diagnostic ability (Figure 4A). The area under the ROC curve for perillic acid, palmitoleic acid, N‐Acetyl‐d‐sphingosine, oleic acid, palmitic acid, 2‐Octenoylcarnitine, alpha‐linolenic acid, arachidonic acid, docosahexaenoic acid and octadecanoic acid was 96%, 89%, 84.5%, 82.8%, 82%, 79.5%, 79.5%, 76% and 75% in CHD‐PAH patients, respectively, which indicated perillic acid had a good diagnostic ability (Figure 4B). The joint diagnostic efficacy of selected metabolites above was analysed by random forest method, and the AUC for IPAH or CHD‐PAH were 97.7%‐98.8% and 95.4%‐96.2%, respectively (Figure 4C,D).

### 3.6 Haemodynamic index of MCT‐induced PAH rats

In the second week (day 14) and the third week (day 21), mean pulmonary arterial pressure (mPAP) (Figure S1A), RVSP (Figure S1B), right ventricular hypertrophy index (RVHI) (Figure S1C) and MT% (external diameter/external diameter) (Figure S1E) of rats in MCT group were significantly higher than that in control group (all *P* < .01). In comparison with the control group, the rats with MCT induction for 2 or 3 weeks displayed a thickened inner wall of the pulmonary artery, hypertrophic intima and tunicae media of pulmonary artery, and an evidently narrowed vascular lumina (Figure S1D).

### 3.7 Changes of genes and proteins involved in glucose and fatty acid metabolism in lungs of MCT‐induced PAH rat model

**Figure 5:**
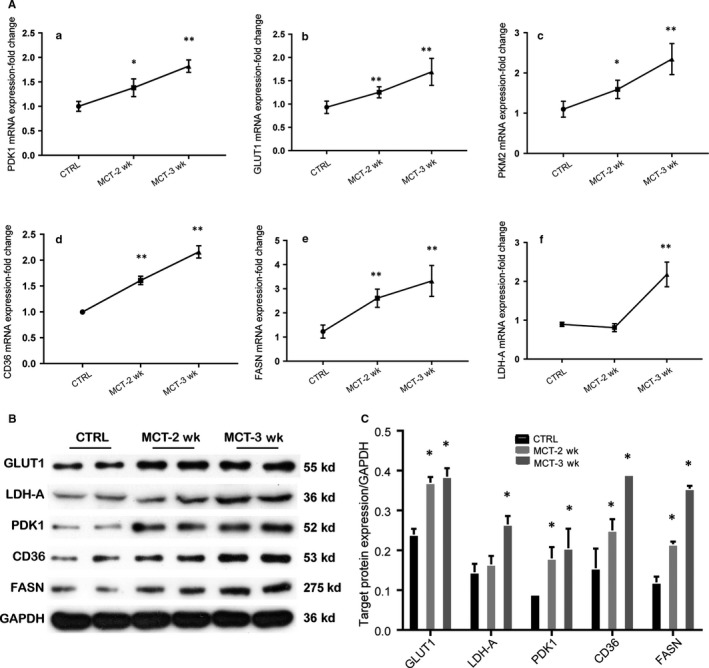
Changes of genes and proteins involved in glucose and fatty acid metabolism in lungs of MCT‐induced PAH rat model. Male SD rats (180 g) randomly received an intraperitoneal injection of normal saline (CTRL, n = 12) or monocrotaline (MCT, n = 24) to induce PAH. The rats in control group were examined at the third week (day 21), and rats in MCT group were randomly examined at the second (day 14, MCT‐2 wk, n = 12) and third week (day 21, MCT‐3 wk, n = 12). The mRNA expressions of PDK1 (a), GLUT1 (b), PKM2 (c), CD36 (d), FASN (e) and LDHA (f) were tested by real‐time PCR (A), relative expression levels of proteins were tested by Western blot (B), relative levels of GLUT1, LDHA, PDK1, CD36 and FASN were calculated (C). *P < .05; **P < .01

We investigated the mRNA and protein expression levels of key enzymes in glucose and fatty acid metabolism in lungs. The results showed the mRNA expression levels of PDK1, GLUT1, PKM2, CD36 and FASN were significantly increased in lungs when rats were treated with MCT for 2 or 3 weeks, and mRNA expression of LDHA was significantly increased only in the third week of MCT treatment, compared with the control group (all *P* < .05) (Figure 5A). There was no significant change in mRNA expression levels of GLUT4, PDK4, LDH2, CPT‐1β and MCD in response to MCT treatment (data not shown). Western blot analyses of protein levels showed similar results, except for that the protein expression level of PKM2 did not alter in response to MCT treatment (Figure 5B,C).

### 3.8 Changes of genes and proteins involved in glucose and fatty acid metabolism in right hearts of MCT‐induced PAH rat model

**Figure 6:**
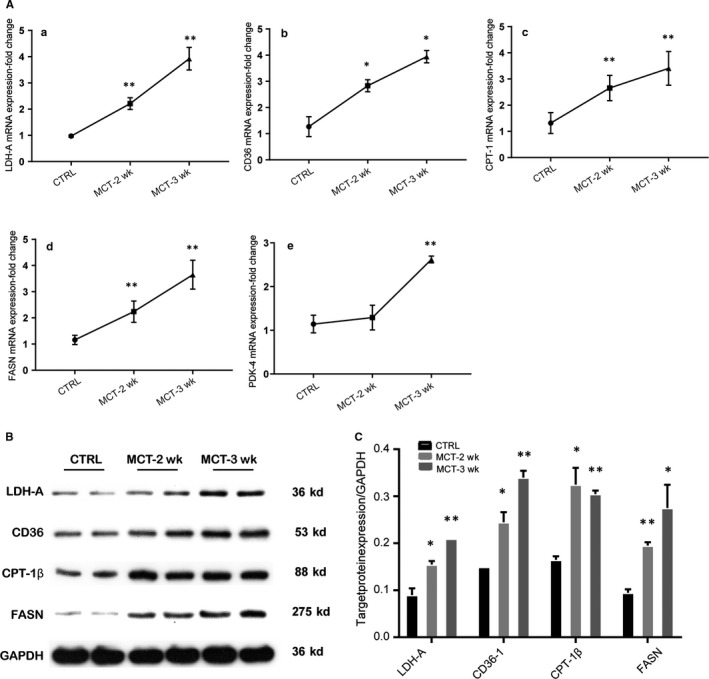
Changes of genes and proteins involved in glucose and fatty acid metabolism in the hearts of the MCT‐induced PAH rat model. Male SD rats (180 g) randomly received an intraperitoneal injection of normal saline (control, n = 12) or monocrotaline (MCT, n = 24) to induce PAH. The rats in control group were examined at the third week (day 21), and rats in MCT group were randomly examined at the second (day 14, MCT‐2 wk, n = 12) and third week (day 21, MCT‐3 wk, n = 12). The mRNA expressions of LDHA (a), CD36 (b), CPT‐1 (c), FASN (d) and PDK4 (e) were tested by real‐time PCR (A), relative protein levels of proteins were tested by Western blot (B), relative levels of LDHA, CD36, cpt‐1β and FASN were calculated (C). *P < .05; **P < .01

We investigated mRNA and protein expression levels of key enzymes in glucose and fatty acid metabolism in lungs. The results showed mRNA expression levels of LDHA, CD36, CPT‐1β and FASN were significantly increased in right heart when rats were treated with MCT for 2 or 3 weeks, and mRNA expression of PDK‐4 was significantly increased only in the third week of MCT treatment, compared with the control group (all *P* < .05) (Figure 6A). Western blot analyses of protein levels of showed similar results, except for that the protein expression level of PDK‐4 did not alter in response to MCT treatment (Figure 6B,C).

## 4 DISCUSSION

Our study provides insight into the metabolic signatures of PAH with the potential to unravel novel biomarkers and therapeutic targets. In our study, the levels of lactic acid in plasma of PAH patients were significantly increased, indicating an increased rate of glycolysis. This has been further corroborated in a study by Rafikova et al^17^ where they found increased level of lactate in lungs of MCT‐induced PAH rats were markedly increased. Similar finding has also been reported by Piao et al^18^ where they report an increased glycolysis in heart of MCT‐induced PAH rats and partial restoration of RV function upon enhancing the oxidative phosphorylation. A possible explanation of our observation can be due to the suppression of glucose oxidative phosphorylation pathway in PAH. Oxidative phosphorylation in the mitochondria is required for upregulation of ATP synthesis in the cell that can resist the cell from undergoing apoptosis and rapid proliferation. Pyruvate dehydrogenase kinase‐1 (PDK‐1), a key enzyme of the glycolytic pathway, is also a HIF1‐regulated protein and induces rapidly proliferation of cells by inhibiting their apoptosis.^19^ The fact that we also observe increased mRNA and protein expression of PDK‐1 in the lungs of MCT‐induced PAH rats, which strengthens our hypothesis of a metabolic basis of PAH development. Subsequently, we found the transcriptional and translational increase in lactate dehydrogenase A (LDHA) in the lungs and hearts of MCT‐induced PAH rats. LDHA is another cytosolic enzyme in the glycolytic pathway that catalyses the interconversion of pyruvate and l‐lactate and aids in the interconversion of NADH and NAD^+^. This effect of LDHA has been extensively studied in in vitro cancer models where tumour cells proliferate under hypoxic condition by suppression of mitochondrial respiration (Warburg effect).^20^ Taken together, targeting the glycolysis and associated enzymes appears to be a lucrative therapeutic strategy in PAH. In this regard, GLUT1 facilitating the transport of glucose across the plasma membranes of cells is of considerable interest. Increase in GLUT1 and subsequent increase in glucose uptake has been implicated in providing a “glycolytic” metabolic profile in the lungs and heart in PAH patients from prior studies.^21^, ^22^, ^23^ This effect was more pronounced in proliferating vascular cells in PAH rats,^22^ and inhibition of GLUT1 expression has been demonstrated to reverse Warburg effect as well,^23^ making it a novel therapeutic target. In our study, we observe marked increase in the expression of GLUT1, as well.

However, the level of citric acid and l‐malic acid, intermediate product of the tricarboxylic acid cycle, were also increased in our PAH patients, in comparison to the healthy controls. It can be due to enhanced mitochondrial fatty acid oxidation by the Randle cycle, as evidenced by the substrate replacement for generation of ATP in these proliferating cells.

In present study, we report that the levels of l‐carnitine, acetyl‐l‐carnitine and several long‐chain acylcarnitines including palmitoylcarnitine, oleoylcarnitine, decenoylcarnitine, linoelaidyl carnitine, cis‐5‐Tetradecenoylcarnitine and trans‐2‐Dodecenoylcarnitine in the peripheral blood of patients with PAH were significantly higher than those of healthy controls. Blocking of β‐oxidation of fatty acid results in large accumulation of acyl‐CoA, and acyl‐CoA can combine with carnitine and then be exported from cell into extracellular fluid, leading to an increase in peripheral circulating acylcarnitine levels. Therefore, increased levels of carnitine and acylcarnitine may reflect the significant inhibition of mitochondrial fatty acid β‐oxidation during the development of PAH. Burgeoning evidence points towards the metabolic homeostasis of carnitine that is integral in the formation and development of PAH.^24^, ^25^ We also found a significant increase in palmitoleic acid and oleic acid levels in peripheral blood of patients with PAH. Levels of plasma palmitic acid are independent of its dietary intake but are regulated by de novo lipogenesis (DNL). Hypoxia can induce HIF‐1α activation in aberrant proliferating cells and inhibit β‐oxidation of long‐chain fatty acids such as palmitic acid, which leads to its massive accumulation.^26^ Given the increased DNL and palmitic acid build‐up in the cells in cancer cells, our findings closely underline the similarities between cancerous proliferation and vascular endothelial proliferation in PAH.^27^ CD36 is the main fatty acid transporter in myocardium and accounts for approximately 70% of fatty acid uptake into contracting cardiomyocytes.^28^ Our study found that CD36 expression was significantly up‐regulated in lungs and hearts of MCT‐induced PAH, which was consistent with previous studies in the field.^29^, ^30^ Upregulation of CD36 expression increases fatty acid uptake resulting in excess lipid supply and subsequent lipid accumulation in lung and heart which can induce cardiac hypertrophy and contractile dysfunction. Studies have found that palmitic acid increases CD36 expression, and palmitoylation results in abnormal lipid accumulation in cells.^31^ Since we had a significant increase in circulating palmitate, the upregulation of CD36 may be a sequelae to the metabolite. In addition, we have found that the levels of 2‐Octenoylcarnitine and 9‐decenoylcarnitine were decreased in the CHD‐PAH patients. Given our limited understanding about these two lipid subtypes, the significance of these lipids in the circulation remains unknown. Future studies can explore the mechanistic basis of these metabolites in the circulation of PAH patients.

Another interesting finding in our study is the significantly reduced tryptophan levels in the CHD‐PAH group, compared to their healthy counterpart. Serotonin, a metabolite of tryptophan, can induce pulmonary artery smooth muscle proliferation, vasoconstriction and microthrombosis and play a key role in the development of PAH.^32^ Therefore, the decrease in tryptophan in our study may be related to the increased conversion of tryptophan to serotonin, which is contributory to the pulmonary artery endothelial proliferation and vascular remodelling. In addition, we found increased levels of l‐phenylalanine in patients with IPAH, compared with healthy controls. This is significant considering the fact that ratio of (phenylalanine/tyrosine) elevation is considered as a biochemical marker for endothelial dysfunction,^33^ emphasizing the endothelial dysfunction in our cohort of PAH patients.

In present study, we found an increase in levels of phosphatidylcholine (PC) and a decrease in levels of lysophosphatidylcholine (LysPC) in the PAH group, compared with that in the healthy controls. However, their role in disease pathogenesis is unclear and is an area of active research.

## 5 CONCLUSIONS

In conclusion, metabolic profiling in a Chinese population indicates towards a metabolic pathogenesis of PAH, highlighting the significance of these metabolites in biomarker discovery and devising therapeutic strategies.

## CONFLICT OF INTEREST

The authors have no conflict of interest to disclose.

## AUTHORS CONTRIBUTION

ZF, SZ, CC and FL (Fei Luo) conceived the idea; CC and FL (Fei Luo) perform all the experiments, PW, YH, JC help with some experiments, CC and FL (Fei Luo) analysed the data and wrote the manuscript; YH, AD, PW, SC, FL (Fei Luo) and XH collected and read the literature and revised the article; FL (Fei Li), SZ, ZF read through and corrected the manuscript. All authors read and approved the final manuscript.

## Data Availability

Raw data will be made available on request.

## References

[1] Farber HW , Miller DP , Poms AD , et al. Five‐year outcomes of patients enrolled in the reveal registry. Chest. 2015;148:1043‐1054. 26066077 10.1378/chest.15-0300

[2] Sutendra G , Michelakis ED . The metabolic basis of pulmonary arterial hypertension. Cell Metab. 2014;19:558‐573. 24508506 10.1016/j.cmet.2014.01.004

[3] Paulin R , Michelakis ED . The metabolic theory of pulmonary arterial hypertension. Circ Res. 2014;115:148‐164. 24951764 10.1161/CIRCRESAHA.115.301130

[4] Rubin LJ . Metabolic dysfunction in the pathogenesis of pulmonary hypertension. Cell Metab. 2010;12:313‐314. 20889122 10.1016/j.cmet.2010.09.006

[5] Brittain EL , Talati M , Fessel JP , et al. Fatty acid metabolic defects and right ventricular lipotoxicity in human pulmonary arterial hypertension. Circulation. 2016;133:1936‐1944. 27006481 10.1161/CIRCULATIONAHA.115.019351 PMC4870107

[6] Zhao Y , Peng J , Lu C , et al. Metabolomic heterogeneity of pulmonary arterial hypertension. PLoS ONE. 2014;9:e88727. 24533144 10.1371/journal.pone.0088727 PMC3923046

[7] Lewis GD , Ngo D , Hemnes AR , et al. Metabolic profiling of right ventricular‐pulmonary vascular function reveals circulating biomarkers of pulmonary hypertension. J Am Coll Cardiol. 2016;67:174‐189. 26791065 10.1016/j.jacc.2015.10.072 PMC4962613

[8] Rhodes CJ , Ghataorhe P , Wharton J , et al. Plasma metabolomics implicates modified transfer RNAs and altered bioenergetics in the outcomes of pulmonary arterial hypertension. Circulation. 2017;135:460‐475. 27881557 10.1161/CIRCULATIONAHA.116.024602 PMC5287439

[9] Galie N , Humbert M , Vachiery JL , et al. 2015 esc/ers guidelines for the diagnosis and treatment of pulmonary hypertension. Rev Esp Cardiol (Engl Ed). 2016;69:177. 26837729 10.1016/j.rec.2016.01.002

[10] Huang YY , Su W , Zhu ZW , et al. Elevated serum HMGB1 in pulmonary arterial hypertension secondary to congenital heart disease. Vascul Pharmacol. 2016;85:66‐72. 27568461 10.1016/j.vph.2016.08.009

[11] Vuckovic D . Current trends and challenges in sample preparation for global metabolomics using liquid chromatography‐mass spectrometry. Anal Bioanal Chem. 2012;403:1523‐1548. 22576654 10.1007/s00216-012-6039-y

[12] Blasco H , Corcia P , Pradat PF , et al. Metabolomics in cerebrospinal fluid of patients with amyotrophic lateral sclerosis: an untargeted approach via high‐resolution mass spectrometry. J Proteome Res. 2013;12:3746‐3754. 23859630 10.1021/pr400376e

[13] Benjamini Y , Hochberg Y . Controlling the false discovery rate: a practical and powerful approach to multiple testing. J Roy Stat Soc. 1995;57:289‐300.

[14] Agard C , Rolli‐Derkinderen M , Dumas‐de‐La‐Roque E , et al. Protective role of the antidiabetic drug metformin against chronic experimental pulmonary hypertension. Br J Pharmacol. 2009;158:1285‐1294. 19814724 10.1111/j.1476-5381.2009.00445.x PMC2782337

[15] Guo Y , Luo F , Zhang X , et al. TPPU enhanced exercise‐induced epoxyeicosatrienoic acid concentrations to exert cardioprotection in mice after myocardial infarction. J Cell Mol Med. 2018;22:1489‐1500. 29265525 10.1111/jcmm.13412 PMC5824362

[16] Luo F , Guo Y , Ruan GY , et al. Combined use of metformin and atorvastatin attenuates atherosclerosis in rabbits fed a high‐cholesterol diet. Sci Rep. 2017;7:2169. 28526884 10.1038/s41598-017-02080-w PMC5438352

[17] Rafikova O , Meadows ML , Kinchen JM , et al. Metabolic changes precede the development of pulmonary hypertension in the monocrotaline exposed rat lung. PLoS ONE. 2016;11:e0150480. 26937637 10.1371/journal.pone.0150480 PMC4777490

[18] Piao L , Fang YH , Parikh K , Ryan JJ , Toth PT , Archer SL . Cardiac glutaminolysis: a maladaptive cancer metabolism pathway in the right ventricle in pulmonary hypertension. J Mol Med (Berl). 2013;91:1185‐1197. 23794090 10.1007/s00109-013-1064-7 PMC3783571

[19] Wigfield SM , Winter SC , Giatromanolaki A , Taylor J , Koukourakis ML , Harris AL . PDK‐1 regulates lactate production in hypoxia and is associated with poor prognosis in head and neck squamous cancer. Br J Cancer. 2008;98:1975‐1984. 18542064 10.1038/sj.bjc.6604356 PMC2441961

[20] Fantin VR , St‐Pierre J , Leder P . Attenuation of LDH‐A expression uncovers a link between glycolysis, mitochondrial physiology, and tumor maintenance. Cancer Cell. 2006;9:425‐434. 16766262 10.1016/j.ccr.2006.04.023

[21] Oikawa M , Kagaya Y , Otani H , et al. Increased [18f]fluorodeoxyglucose accumulation in right ventricular free wall in patients with pulmonary hypertension and the effect of epoprostenol. J Am Coll Cardiol. 2005;45:1849‐1855. 15936618 10.1016/j.jacc.2005.02.065

[22] Marsboom G , Wietholt C , Haney CR , et al. Lung (1)(8)f‐fluorodeoxyglucose positron emission tomography for diagnosis and monitoring of pulmonary arterial hypertension. Am J Respir Crit Care Med. 2012;185:670‐679. 22246173 10.1164/rccm.201108-1562OC PMC3326289

[23] Zhang TB , Zhao Y , Tong ZX , Guan YF . Inhibition of glucose‐transporter 1 (glut‐1) expression reversed warburg effect in gastric cancer cell mkn45. Int J Clin Exp Med. 2015;8:2423‐2428. 25932183 PMC4402830

[24] Sharma S , Sud N , Wiseman DA , et al. Altered carnitine homeostasis is associated with decreased mitochondrial function and altered nitric oxide signaling in lambs with pulmonary hypertension. Am J Physiol Lung Cell Mol Physiol. 2008;294:L46‐L56. 18024721 10.1152/ajplung.00247.2007 PMC3970936

[25] Sharma S , Aramburo A , Rafikov R , et al. l‐carnitine preserves endothelial function in a lamb model of increased pulmonary blood flow. Pediatr Res. 2013;74:39‐47. 23628882 10.1038/pr.2013.71 PMC3709010

[26] Huang LT , Li X , et al. HIF‐1‐mediated suppression of acyl‐coa dehydrogenases and fatty acid oxidation is critical for cancer progression. Cell Rep. 2014;8:1930‐1942. 25242319 10.1016/j.celrep.2014.08.028

[27] Vander Heiden MG , Cantley LC , Thompson CB . Understanding the Warburg effect: the metabolic requirements of cell proliferation. Science. 2009;324:1029‐1033. 19460998 10.1126/science.1160809 PMC2849637

[28] Habets DD , Coumans WA , Voshol PJ , et al. AMPK‐mediated increase in myocardial long‐chain fatty acid uptake critically depends on sarcolemmal CD36. Biochem Biophys Res Comm. 2007;355:204‐210. 17292863 10.1016/j.bbrc.2007.01.141

[29] Talati M , Hemnes A . Fatty acid metabolism in pulmonary arterial hypertension: role in right ventricular dysfunction and hypertrophy. Pulm Circ. 2015;5:269‐278. 26064451 10.1086/681227 PMC4449237

[30] Talati MH , Brittain EL , Fessel JP , et al. Mechanisms of lipid accumulation in the bone morphogenetic protein receptor type 2 mutant right ventricle. Am J Respir Crit Care Med. 2016;194:719‐728. 27077479 10.1164/rccm.201507-1444OC PMC5027228

[31] Lee YS , Kim SY , Ko E , et al. Exosomes derived from palmitic acid‐treated hepatocytes induce fibrotic activation of hepatic stellate cells. Sci Rep. 2017;7:3710. 28623272 10.1038/s41598-017-03389-2 PMC5473841

[32] MacLean MR , Herve P , Eddahibi S , Adnot S . 5‐hydroxytryptamine and the pulmonary circulation: receptors, transporters and relevance to pulmonary arterial hypertension. Br J Pharmacol. 2000;131:161‐168. 10991906 10.1038/sj.bjp.0703570 PMC1572323

[33] Rygula A , Pacia MZ , Mateuszuk L , et al. Identification of a biochemical marker for endothelial dysfunction using Raman spectroscopy. Analyst. 2015;140:2185‐2189. 25664353 10.1039/c4an01998a

