# Supplementary figures and images for: Metabolomics reveals metabolite changes of patients with pulmonary arterial hypertension in China
Source: J Cell Mol Med. 2020 Jan 16;24(4):2484–96. doi: 10.1111/jcmm.14937 (PMC7028857; doi:10.1111/jcmm.14937)

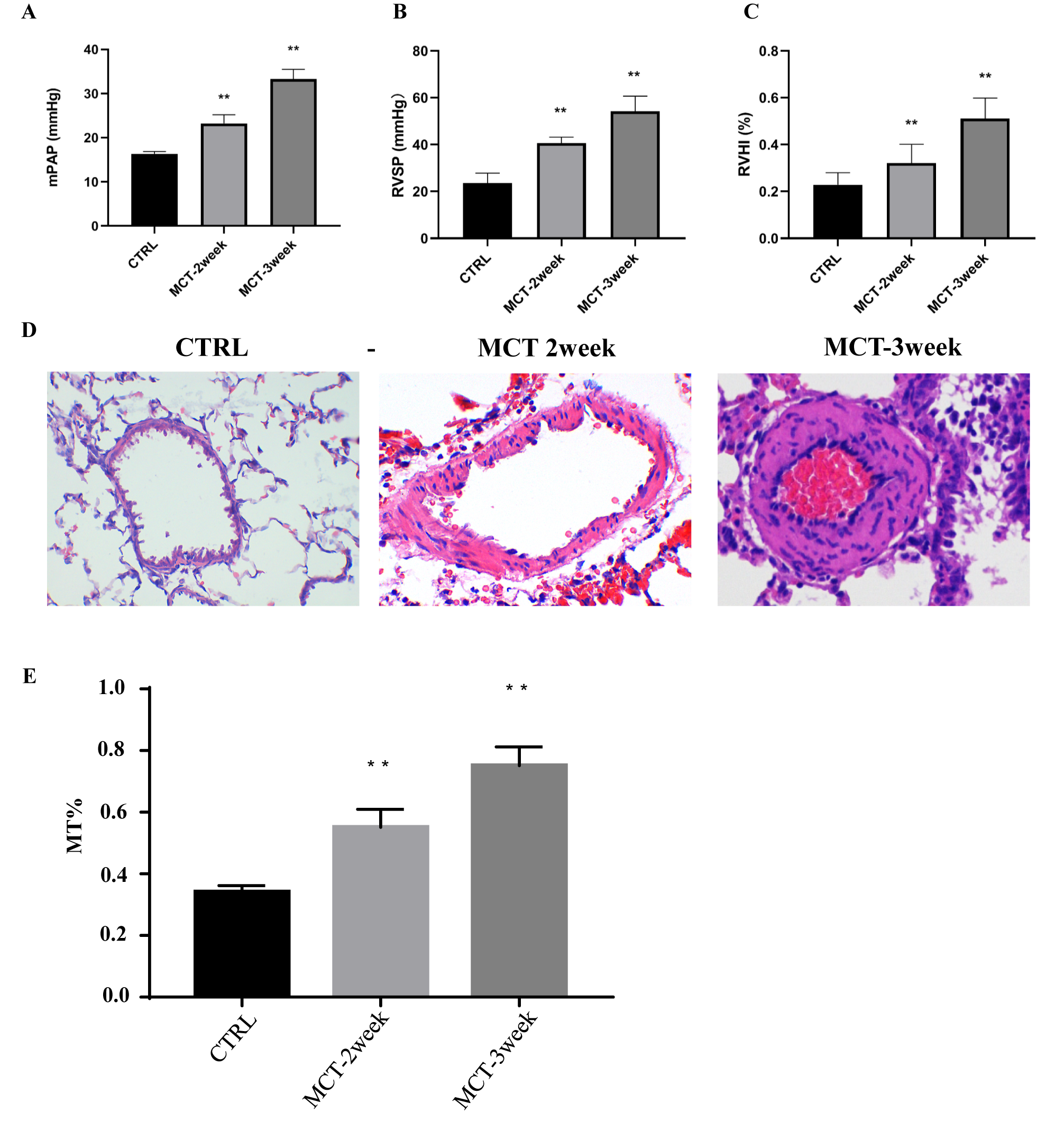

Supplement: Supplementary file 1 [file JCMM-24-2484-s001.tif]
