# Supplementary material for: Metabolomics reveals metabolite changes of patients with pulmonary arterial hypertension in China
Source: J Cell Mol Med. 2020 Jan 16;24(4):2484–96. doi: 10.1111/jcmm.14937 (PMC7028857; doi:10.1111/jcmm.14937)
Supplement: Supplementary file 2 [file JCMM-24-2484-s002.docx]

**Table S1** **parameters of prediction ability of OPLS-DA model**

|  | R2X | R2Y | Q2 | P values |
| --- | --- | --- | --- | --- |
| **Positive ion model** |  |  |  |  |
| IPAH vs Control | 0.67 | 0.91 | 0.77 | 3.8222×10^-6^ |
| CHD-PAH vs Control | 0.71 | 0.89 | 0.73 | 0.0173 |
| IPAH vs CHD-PAH | 0.64 | 0.78 | 0.65 | 0.0412 |
| **Negative ion model** |  | | | |
| IPAH vs Control | 0.65 | 0.9 | 0.83 | 3.3158×10^-9^ |
| CHD-PAH vs Control | 0.71 | 0.89 | 0.74 | 0.0013 |
| IPAH vs CHD-PAH | 0.53 | 0.77 | 0.56 | 0.0036 |

**Table S2** **Metabolites profiles of participants**

|  | Name | RT | Ionization | MS | VIP | *P* value | FDR | Change vs. control | HMDB | |
| --- | --- | --- | --- | --- | --- | --- | --- | --- | --- | --- |
|  | **IPAH vs Control** |  |  |  |  |  |  |  |  | |
| 1 | Palmitoylcarnitine | 10.3 | Positive | 399.334 | 1.19 | 0.0043 | 0.00052 | 2.17 | HMDB0000222 | |
| 2 | Oleoylcarnitine | 10.6 | Positive | 425.349 | 1.97 | < 0.0001 | 0.000051 | 2.17 | HMDB0005065 | |
| 3 | LysoPC(18:2(9Z,12Z) | 10.2 | Positive | 519.331 | 10.2 | < 0.0001 | 0.0131 | 0.63 | HMDB0010386 | |
| 4 | LysoPC(20:4/0:0) | 10.3 | Positive | 543.33 | 3.06 | 0.00359 | 0.0203 | 0.73 | HMDB0010396 | |
| 5 | L-Carnitine | 0.83 | Positive | 161.105 | 3.17 | 0.02019 | 0.0280 | 1.23 | HMDB0000062 | |
| 6 | Acetyl-L-carnitine | 1.2 | Positive | 203.115 | 1.22 | 0.00039 | 0.00179 | 1.51 | | HMDB0000201 |
| 7 | (C10) Decanoylcarnitine | 7.28 | Positive | 315.24 | 1.26 | 0.00968 | 0.0232 | 1.34 | | HMDB0000651 |
| 8 | L-Threonic acid | 0.98 | Positive | 136.036 | 4 | 0.00863 | 0.0032 | 3.91 | | HMDB0062620 |
| 9 | L-Phenylalanine | 0.98 | Positive | 165.08 | 2.19 | 0.035 | 0.09 | 1.55 | | HMDB0000159 |
| 10 | Geranyl-PP | 0.86 | Positive | 314.07 | 1.68 | 0.0316 | 0.0807 | 0.87 | | HMDB0001285 |
| 11 | N-Acetyl-D-sphingosine | 14.5 | Positive | 341.291 | 1.02 | 0.02168 | 0.0205 | 3.31 | | Database |
| 12 | Creatine | 0.85 | Positive | 131.069 | 1.61 | 0.04131 | 0.0557 | 0.72 | | HMDB0000064 |
| 13 | trans-2-Dodecenoylcarnitine | 7.77 | Positive | 341.257 | 1.14 | 0.00073 | 0.000544 | 2.26 | | HMDB0013326 |
| 14 | cis-5-Tetradecenoylcarnitine | 8.76 | Positive | 369.287 | 1.27 | 0.0022 | 0.00155 | 2.17 | | HMDB0002014 |
| 15 | Linoelaidyl carnitine | 9.93 | Positive | 423.334 | 1.08 | 0.00466 | 0.00542 | 1.58 | | HMDB0006461 |
| 16 | Linoleoyl ethanolamide | 14.5 | Positive | 323.281 | 1.6 | 0.02022 | 0.0192 | 3.20 | | HMDB0012252 |
| 17 | PC(16:0/20:5) | 13.8 | Positive | 779.546 | 4.93 | 0.03173 | 0.0207 | 1.27 | | HMDB0007984 |
| 18 | Palmitic acid | 14.4 | Negative | 256.237 | 1.66 | 0.01094 | 0.0179 | 1.34 | | HMDB0000220 |
| 19 | Oleic acid | 14.5 | Negative | 282.256 | 4.49 | 0.00274 | 0.0036 | 1.98 | | HMDB0000207 |
| 20 | Docosahexaenoic acid | 13.4 | Negative | 328.237 | 1.6 | 0.00616 | 0.0114 | 1.85 | | HMDB0002183 |
| 21 | Palmitoleic acid | 13.5 | Negative | 254.221 | 1.83 | 0.01029 | 0.0118 | 4.32 | | HMDB0003229 |
| 22 | D-Lactic acid | 0.99 | Negative | 90.033 | 10.1 | 0.00042 | 0.0025 | 1.79 | | HMDB0001311 |
| 23 | Citric acid | 0.98 | Negative | 192.026 | 2.83 | 0.00049 | 0.0130 | 1.52 | | HMDB0000094 |
| 24 | L-Malic acid | 0.91 | Negative | 134.021 | 1.14 | < 0.0001 | < 0.0001 | 2.53 | | HMDB0000156 |
| 25 | L-1-Pyrroline-3-hydroxy-5-carboxylate | 0.99 | Negative | 129.042 | 1.48 | 0.02037 | 0.025 | 1.45 | | HMDB0001369 |
| 26 | Perillic acid | 8.53 | Negative | 166.099 | 1.19 | < 0.0001 | 0.00081 | 0.33 | | HMDB0004586 |
|  | **CHD-PAH vs Control** |  |  |  |  |  |  |  | |  |
| 1 | L-Carnitine | 0.83 | Positive | 161.105 | 5.09 | 0.00407 | 0.0505 | 1.40 | | HMDB0000062 |
| 2 | N-Acetyl-D-sphingosine | 14.5 | Positive | 341.291 | 1.68 | 0.00041 | 0.01124 | 4.68 | | Database |
| 3 | Linoleoyl ethanolamide | 14.5 | Positive | 323.281 | 2.63 | 0.00035 | 0.0106 | 4.59 | | HMDB0012252 |
| 4 | 2-Octenoylcarnitine | 5.56 | Positive | 285.194 | 1.69 | 0.00019 | 0.00973 | 0.61 | | HMDB0013324 |
| 5 | 9-Decenoylcarnitine | 6.83 | Positive | 313.225 | 1.01 | 0.04226 | 0.0509 | 0.73 | | HMDB0013205 |
| 6 | Palmitic acid | 14.4 | Negative | 256.237 | 2.29 | 0.00101 | 0.0203 | 1.623 | | HMDB0000220 |
| 7 | Oleic acid | 14.5 | Negative | 282.256 | 5.7 | < 0.0001 | 0.00628 | 2.57 | | HMDB0000207 |
| 8 | Docosahexaenoic acid | 13.4 | Negative | 328.237 | 1.85 | 0.01333 | 0.0579 | 2.60 | | HMDB0002183 |
| 9 | Palmitoleic acid | 13.5 | Negative | 254.221 | 2.04 | 0.00141 | 0.0394 | 5.56 | | HMDB0003229 |
| 10 | D-Lactic acid | 0.99 | Negative | 90.033 | 4.77 | 0.02632 | 0.0997 | 1.68 | | HMDB0001311 |
| 11 | L-Tryptophan | 3.31 | Negative | 204.088 | 1.04 | 0.02922 | 0.0716 | 0.83 | | HMDB0013609 |
| 12 | Alpha-Linolenic acid | 13.1 | Negative | 278.221 | 2.98 | 0.00414 | 0.0391 | 5.11 | | HMDB0001388 |
| 13 | Arachidonic acid | 13.6 | Negative | 304.237 | 2.42 | 0.00868 | 0.0534 | 2.94 | | HMDB0001043 |
| 14 | Perillic acid | 8.53 | Negative | 166.099 | 1.11 | < 0.0001 | 0.0014 | 0.24 | | HMDB0004586 |
| 15 | Octadecanoic acid | 15.6 | Negative | 284.272 | 1.43 | 0.03 | 0.0707 | 1.26 | | HMDB0000827 |

**Supplementary Figure S1 Haemodynamic index of MCT-induced PAH rats.**

Male SD rats (180g) randomly received an intraperitoneal injection of normal saline (CTRL, n=12) or monocrotaline (MCT, n=24) to induce PAH. The rats in control group were examined at the third week (day 21), and rats in MCT group were randomly examined at the second (day 14, MCT-2week, n=12) and third week (day 21, MCT-3week, n=12). Values of mPAP (A), RVSP (B), and RVHI (C) are presented. Rat lung tissues involved with the CTRL, MCT-2week and MCT-3week groups were placed with 10% formalin, embedded at 4℃ in paraffin and sliced to a thickness of 4 μm. The sections were performed with H&E stain, and their histological changes were observed under an optical microscope (D). Medial thickness (MT), external diameter (ED) were measured, the ratios of MT/ED (MT%) are calculated (E). **, P< 0.01. mPAP: mean pulmonary arterial pressure, RVSP: ventricular systolic pressure, RVHI: right ventricular hypertrophy index.
